# Treatment of Pneumonia

**Published:** 1857-07

**Authors:** C. J. Pope

**Affiliations:** Eufaula, Alabama


ART. IV.—*Treatment of Pneumonia.* By C. J. POPE, M.D., of Eufaula, Alabama.

IN view of the wide-spread prevalence of pneumonia in this section of country during the past winter and spring, the probabilities of its recurrence under similar circumstances, and the great fatality which has generally marked its progress, I am induced to state to the profession the mode by which I have so far this season successfully treated every case that has fallen under my care. The number of cases thus treated, was not less than forty, which in a community such as that in which I practise, is an unusually large experience for one individual during a single season. Of the causes which probably gave rise to this epidemic, unprecedented in this neighborhood, I can refer only to the intensely cold and protracted winter.

*Treatment*—When the onset of the disease is so violent that the system reacted tardily, a circumstance which I have several times observed, I administered brandy with a more unsparing hand than I ever dared to do in any previous season, and have often been obliged to continue its use for several hours. And in such cases, I may here remark—cases in which the vital energies are so overpowered by the disease that reaction was slow in being established—I have never found it necessary to take blood. When, however, reaction is prompt, whether unaided or otherwise, I invariably bled once, and once only, but generally freely. Bleeding having been premised, I put the patient upon the use of opium and calomel, in doses of from one to one and a half grains of the former, according to the intensity of the pain and severity of the cough, and from two to four grains of the latter, to be taken every three hours until three or four were taken. Waiting then from one to three hours, I administered a dose of castor oil, which having produced the desired effect, I returned to the use of the opium, giving it in the same doses and at the same intervals as before, but combined now with the tincture of veratrum viride in doses of about six drops. This I continued until there was a marked impression on the circulation, when I diminished the proportion of the veratrum, but not that of the opium, until at least twenty-four hours had elapsed since the commencement of its use. If at the end of that time the disease became evidently arrested, which was generally the case, I lessened the dose of the opium, or what was decidedly better, gave it at longer intervals. I found that in thirty-six or forty-eight hours from the time the opium and veratrum viride were first given, the case was generally cured, so far as the inflammatory action was concerned, and farther treatment unnecessary.

When called to a case which has not received the prompt and efficient treatment just mentioned, I have often found it necessary to cup and blister, but as a general rule this practice may be dispensed with, and hot poultices to the chest substituted in their stead.

I am aware that the administration of opium in large doses, in the commencement of an inflammatory disease, is at variance with the generally received notion of the medical profession with regard to this medicine, nor am I prepared to deny with positiveness the soundness of this opinion, when the opium is given alone; but in conjunction with veratrum viride, its action is as certainly sedative and diaphoretic as it is anodyne, and, therefore, when thus given, it is unquestionably admissible. I regard the conjoined action of these two agents as the *desideratum* in the treatment of pneumonia. For the purpose of encouraging the perspiration, which is induced by these remedies, I administer the tea of the asclepias tuberosa, and consider it a valuable adjuvant.

I have just stated that I am not prepared to assert with positiveness that opium does not act as a stimulant in the early stages of inflammatory diseases, and that it is on that account contraindicated, but I confess that my decided convictions are to the contrary, at least where it is given in pneumonia, where the pain is intense, and cough excessive, provided the doses are sufficiently large to exert a marked control over the pain. This being accomplished, the circulation will also be controlled, and the determination to the point of excitement in the lungs will be diverted towards the surface, and in a large majority of cases, diaphoresis will be the result.

I have not administered the opium alone, under such circumstances, for the reason that I have always at hand a remedy which, when given in conjunction with it, effects all that I could ask in controlling this formidable disease, and I have not felt warranted in departing from a course which was sure of success, for one around which hung at least a shadow of doubt. I repeat, that if opium, administered by itself in decided doses, will, in this disease, and under the circumstances mentioned above, accelerate the circulation and do mischief, if it is administered in conjunction with the veratrum viride, the latter will obtain the mastery, and their conjoined action will be sedative and diaphoretic in a marked degree, and therefore salutary beyond all doubt. Under this plan of treatment, I

regard the cure as certain and rapid, and failure next to impossible; and if success were not complete, I should look for the cause of failure not in the remedies, but in the mode of administering them.

